# Clinical and Technical Considerations of an Open Access Telehealth Network in South Carolina: Definition and Deployment

**DOI:** 10.2196/17348

**Published:** 2020-05-22

**Authors:** Kathryn King, Dee Ford, Michael Haschker, Jillian Harvey, Ryan Kruis, James McElligott

**Affiliations:** 1 Department of Pediatrics Medical University of South Carolina Charleston, SC United States; 2 Department of Pulmonary and Critical Care Medicine Medical University of South Carolina Charleston, SC United States; 3 Center for Telehealth Medical University of South Carolina Charleston, SC United States; 4 Department of Healthcare Leadership and Management Medical University of South Carolina Charleston, SC United States

**Keywords:** telehealth, health information technology

## Abstract

**Background:**

Today, telehealth is experiencing exponential growth in utilization. Paralleling this trend is the growth in the telehealth industry, with sharp increases in the number of platforms, functionalities, and levels of integrations within both the electronic health record and other technical systems supporting health care. When a telehealth network is intended to be used across independent health care systems, an additional layer of complexity emerges. In the context of regionalized telehealth networks that are not within the same health care system, not only are technical interoperability challenges a practical barrier, but administrative, clinical, and competitive elements also quickly emerge, resulting in fragmented, siloed technologies.

**Objective:**

The study aimed to describe a statewide approach to deploying an interoperable open access telehealth network across multiple health systems.

**Methods:**

One promising solution to the abovementioned concerns is an open access telehealth network. In the field of telehealth, an open access network (OAN) can be defined as a network infrastructure that can be used by health care providers without a closed or proprietary platform, specific obligatory network, or service-specific telehealth technologies. This framework for the development of an OAN is grounded in practical examples of clinical programs that function in each stage of network maturity based on the experience of the South Carolina Telehealth Alliance (SCTA). The SCTA’s experience details successes and challenges in an ongoing effort to achieve an OAN. The model describes an OAN in stages of collaborative maturity and provides insights into the technological, clinical, and administrative implications of making the collaboration possible.

**Results:**

The four stages of an OAN are defined according to operational maturity, ranging from feasibility to demonstration of implementation. Each stage is associated with infrastructure and resource requirements and technical and clinical activities. In stage 1, technical standards are agreed upon, and the clinical programs are designed to utilize compliant technologies. In stage 2, collaboration is demonstrated through technical teams working together to address barriers, whereas clinical and administrative teams share best practices. In stage 3, a functional interoperable network is demonstrated with different institutions providing service through common telehealth end points at different patient care sites. In stage 4, clinical workflows are streamlined and standardized across institutions, and economies of scale are achieved through technical and administrative innovations.

**Conclusions:**

The approach to OAN development described provides a roadmap for achieving a functional telehealth network across independent health systems. The South Carolina experience reveals both successes and challenges in achieving this goal. The next steps toward the development of OANs include advocacy and ongoing engagement with the developers of telehealth technologies regarding their commitment to interoperability.

## Introduction

### Background

Today, telehealth is experiencing exponential growth in utilization [1,2]. Paralleling this trend is the growth in the telehealth industry, with sharp increases in the number of platforms, functionalities, and levels of integrations within both the electronic health record (EHR) and other technical systems supporting health care. When a telehealth network is intended to be used across independent health care systems, an additional layer of complexity emerges. In the context of regionalized telehealth networks that are not within the same health care system, not only are technical interoperability challenges a practical barrier, but administrative, clinical, and competitive elements also quickly emerge, resulting in fragmented, siloed technologies. These observations echo those of the rapid deployment of EHRs that led to fragmentation and barriers to interoperability across systems that have become a practical issue and barrier to optimal care for patients, providers, and health systems [3]. As rapid deployment of telehealth technology follows a similar trajectory, it becomes increasingly important to learn from the EHR example and develop roadmaps for telehealth collaborative solutions before siloed technology becomes standard practice.

### Open Access Network

One promising solution to these concerns is an open access telehealth network. In the field of telehealth, an open access network (OAN) can be defined as a network infrastructure that can be used by health care providers without a closed or proprietary platform, specific obligatory network, or service-specific telehealth technologies. An OAN functioning at the highest level of maturity would also include clinical and administrative workflow standardization. Many manufacturers offer turnkey solutions for starting telehealth programs by selling closed systems and/or proprietary technologies. Although these solutions address immediate needs, they also create closed or siloed networks that cannot be easily accessed or expanded and lack interoperability with other telehealth solutions.

An OAN benefits a health care system as a whole by mitigating the need for proprietary equipment and the specialized staff and contracts that support such equipment. In addition, individual institutions and broader regional health care systems benefit from an OAN as they are able to connect to a more extensive array of subspecialty providers using the same technology, thus saving space, cost, and time in the deployment of equipment. Finally, both providers and patients benefit from familiarity with standardized equipment, increasing adoption for these pivotal end user groups. Open platforms ensure that additional consideration is given to maintaining reasonable costs for broad participation among regional health systems.

Similar to the experience of EHR development, the need for telehealth interoperability was acknowledged nearly a decade ago [4], though the reality of interoperability and effectiveness are not well described [5]. Although the development of an interoperable telehealth network is less complex than a full health information exchange, there are many parallels to consider. The National Quality Forum introduced measures for health information exchange interoperability (Table 1), and the majority of measures go beyond whether the systems are technically interoperable and instead focus on whether the system is used as intended and if that use is effective [5]. In much the same way, a telehealth network may be built to be interoperable, but health systems may choose to selectively deploy this function. For instance, these systems are likely to make variable investments in their own telehealth efforts that are reflective of their variable business cases for making technical decisions. It should be no surprise then that during the growth of telehealth there may also be the need to focus on metrics that relate to interoperability. In this paper, a statewide approach to deploying an interoperable OAN across multiple health systems is described.

**Table 1:** National Quality Forum domains and subdomains of interoperability.

Domain	Subdomains
Exchange of EHI^a^	Availability of EHI Quality of data Method of exchange
Usability of exchanged EHI	Relevance Accessibility Comprehensibility
Application of exchanged EHI	Human use Computable
Impact of interoperability	Patient safety Cost savings Productivity Care coordination Improved health care processes and health outcomes Patient/caregiver engagement Patient/caregiver experience

^a^EHI: electronic health information.

## Methods

The framework for the development of an OAN is grounded in practical examples of clinical programs that function in each stage of network maturity. Although the model is intended to be generalizable, the experience of the South Carolina Telehealth Alliance (SCTA) is used as a representative use case. The SCTA was established in 2013 as the product of legislatively appropriated state support for telehealth in South Carolina. The multistakeholder alliance includes representatives from local health care systems and payers and has a mission to support the delivery of high-value telehealth across the state. Across South Carolina’s 46 counties, there are currently over 400 sites equipped for telehealth services. The SCTA’s experience details successes and challenges in an ongoing effort to achieve an OAN. The model presented here describes an OAN in stages of collaborative maturity and provides insights into the technological, clinical, and administrative implications of making the collaboration possible.

## Results

### Developmental Stages of the Open Access Network

**Figure 1:**
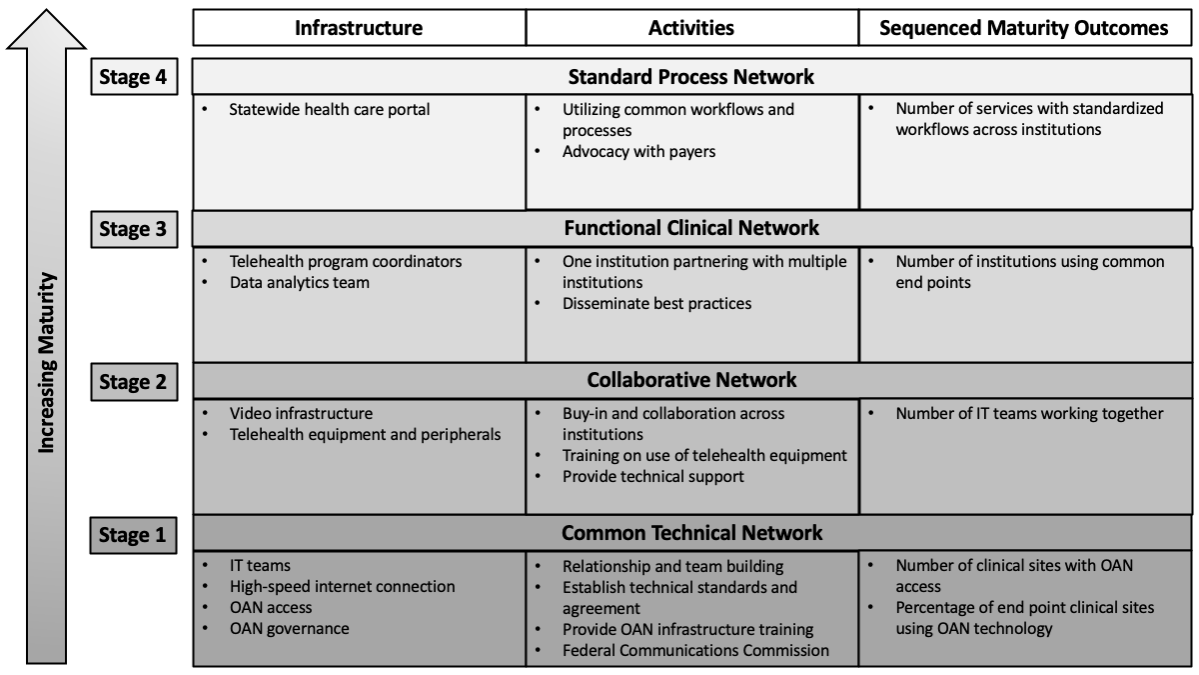
Proposed model for maturing a telehealth open access network. FCC: Federal Communications Commission; IT: information technology; OAN: open access network.

In South Carolina, a vision for technologic interoperability was and remains a key strategy of the state-sponsored telehealth network [6]. The aspirational goal is to allow access to all providers wishing to leverage any deployed telehealth technology in the state. Although achieving this goal is an ongoing challenge, South Carolina has made substantial progress and learned important lessons. A four-stage process is being used to develop and mature a statewide OAN (Figure 1). These four stages are defined according to operational maturity, ranging from feasibility to demonstration of implementation. Each stage is associated with infrastructure and resource requirements, technical, and clinical activities. In stage 1, technical standards are agreed upon, and the clinical programs are designed to utilize compliant technologies. In stage 2, collaboration is demonstrated through technical teams working together to address barriers, whereas clinical and administrative teams share best practices. In stage 3, a functional interoperable network is demonstrated with different institutions providing service through common telehealth end points at different patient care sites. In the fourth and final stage, clinical workflows are streamlined and standardized across institutions, and economies of scale are achieved through technical and administrative innovations (eg, common scheduling portals and standard contract language; Figure 1).

#### Stage 1: Establish a Common Technical Network

##### Establish a Collaborative Forum

As in all collaborative efforts that involve multiple stakeholders, establishing a forum for communication, discussion, and gaining trust among the parties is essential. These stakeholders are likely to be asked to accommodate some adjustments internally for the good of the network; thus, it is important to involve them at early stages of strategic planning and the setting of short- and long-term goals. In South Carolina, the SCTA engages stakeholders with an advisory council and a system of workgroups that operate under the alignment of a formal strategic plan [6]. These workgroups, made up of personnel from the various involved institutions, are responsible for operationalizing the plan within their respective organizations and reporting back to the advisory council. The SCTA information technology (IT) workgroup has become a common ground for members to build relationships and make collaborative decisions with the common goal of interoperability, scalability, and support of the network.

##### Establish Technical Standards and Agreement

As discussed, the inception of a regional telehealth network requires careful planning and coordination of efforts. Defining network requirements and functionality helps drive important decisions on technical protocols and the types of equipment used. The backbone of the OAN in South Carolina is a video network whose interoperability is made possible by using standardized specifications. These specifications are based upon open standards as defined by the International Telecommunications Union (ITU) and via a collaborative approach defined by the IT workgroup within the SCTA [7]. ITU open standards address specific requirements for functionality, interoperability, and compatibility. Through the utilization of existing infrastructure, the network leveraged investment protection while allowing sites and health care providers choices in the platforms they would use if they met the ITU open systems standards.

##### Assess and Address Broadband Availability

**Figure 2:**
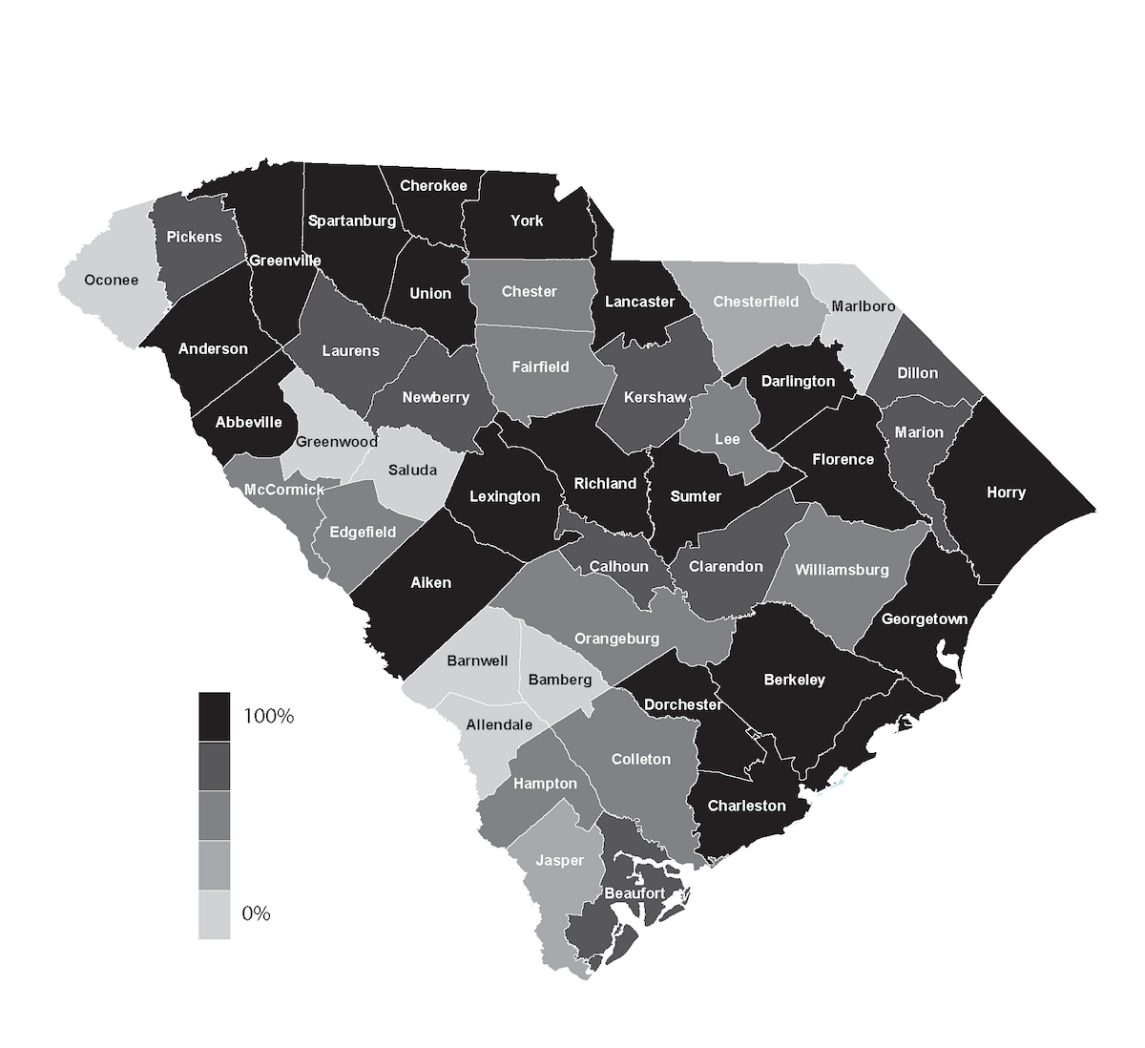
Federal Communications Commission broadband availability in South Carolina.

In South Carolina, rural health care providers often lack technical support, person power, and adequate broadband access. This limited connectivity compounds existing health care access issues, particularly in a region that is mostly rural (Figure 2). Although broadband access is beyond the scope of this paper, it is an important element to address when considering an OAN. When South Carolina designed and implemented a statewide telehealth network, one of the primary requirements was the ability to be used across rural providers and independent health care systems. Many of these health care systems had existing infrastructure and isolated networks that could be reconfigured to work together as one large statewide telehealth network. To build the network, South Carolina utilized the Federal Communications Commission’s (FCC) rural health care program that subsidized infrastructure and broadband connectivity, effectively creating a dedicated statewide health care network. The network is known as the Palmetto State Providers Network (PSPN) [8]. Today, PSPN has over 300 participating health care sites providing affordable low-latency dedicated bandwidth with access to secure encrypted video infrastructure for the delivery of telehealth applications and services.

##### Open Access Network User End Points

Telehealth end points are hardware platforms that users control to establish connections between referring and consulting telehealth participants. An end point is where physical and logical connections to video and audio systems such as cameras, speakers, and microphones are made. End points are the *end of the line* for the video conferencing connection and are different from other components of a video conferencing network such as gateways or multipoint conference units (bridges), which are video infrastructure components.

End points used in telehealth settings must be dependable and easy to use and meet the needs of the patient and health care provider. Under the OAN system, the patient and provider are not limited to consultations within a specific platform. There are many platforms/end point choices that promote technological efficiencies and streamlined patient care. End points come in a number of platforms and include software and PC-based, tablets, wall-mounted hardware-based units, dedicated desktop units, and the popular clinical cart units widely used in hospital settings. In health care facilities where space and resources are at a premium, the ability to utilize end points across multiple telehealth services and platforms is essential to the growth of the telehealth provision and utilization. The South Carolina approach to end point decisions is to educate providers on what is available, listen to the provider’s needs, and then recommend the best solution. Ultimately, it is the health care provider’s decision on what is the best end point for the delivery of health care services.

##### Open Access Network Technical Support

With the deployment of health care networks that provide services for critical and acute care patients, technical reliability is mandatory. Choosing industry-proven commercial-grade network equipment is an essential component for reliability. Having service-level agreements with the manufacturers and providers of network equipment is also a critical element to building and operating a strong network. Knowing that all equipment fails over time, managing equipment life cycles, and providing routine scheduled maintenance and upgrades are essential in maintaining a robust telehealth network. Networks built with designed redundancy or high availability offer both hardware protection and the ability to perform maintenance and upgrades anytime, day or night. Utilizing site and service agreements, the SCTA offered a collaborative approach to technical support where the regional health care systems provide technical and site support to rural providers and smaller clinics in their region.

##### Open Access Network and Telehealth Software

One primary question faced when telehealth software is applied within an OAN is whether the software restricts the type of end point that can be utilized. A key component of this question is whether the telehealth end points not manufactured by the vendor can be used when the vendor’s software initiates the video call. In addition, there are multiple questions that should be collaboratively discussed and considered before selecting telehealth software (Table 2). Conversely, it is important to consider whether the end points that are manufactured by the vendor can be utilized without the vendor’s software. Many national telehealth vendors are moving toward interoperability with computers and tablet-based devices, and many video conferencing products can be configured to call video solutions across a range of video clients. When applying telehealth software, which leverages or augments video conferencing, there are typically two broad uses: encounter management and video network management. Encounter management involves assistance with the workflows of the clinical process; this may include gathering of data to assist clinical decision making, such as radiological imaging to assist in the assessment of a stroke. Encounter assistance may also include administrative functions of care, such as scheduling, facilitating collaborative documentation between care teams, or providing information for billing purposes. However, the functionality of the end points or the software remains constricted by certain specifications determined by telehealth software and equipment manufacturers. For instance, many telehealth encounters are facilitated with the use of far-end camera control in which the distant provider can simulate the in-person experience by controlling the camera on the patient’s side. The ability to leverage this functionality may depend on the functionality of telehealth support software or on the specific camera included in the end point. Restrictions in functionality may also be experienced for the use of examination peripherals, which exclude some services from being offered as the appropriate standard of care.

**Table 2:** Questions to consider when evaluating technologies for users of the open access network.

Category	Questions
Infrastructure	What problem are we solving? What are the current video client- or cloud-based services in use? Does the technology require changes to existing OAN^a^ infrastructure? Does the technology require additional infrastructure?
OAN standards/compatibility	Will functionality be lost when crossing between software and certain standards-based endpoints in the network? Specifically: Far-end camera control Peripheral examinations devices including examination cameras and stethoscopes Are there any end points that software apps provided by a vendor cannot connect to via video? Are the new technologies compatible with the OAN?
Technical support/outcomes	What are the technical support requirements? Is there the ability to monitor the end points to ensure they are online when clinically needed? Is the technology able to provide feedback to monitor key process metrics and data to support relevant quality and outcome measures?
Legal/compliance	What are the state and federal regulatory requirements (including Health Insurance Portability and Accountability Act, Family Educational Rights and Privacy Act, and Commission on Accreditation for Law Enforcement Agencies)? What are the information security requirements addressing the use of video technologies in health care settings? What end point encryption is required?

^a^OAN: open access network.

The process of selecting technology for use within the OAN requires careful planning to ensure interoperability and appropriateness for each use case. Table 2 outlines several categories of questions that may be helpful to consider in this process.

##### Stage 1 Example: Connecting Medication-Assisted Therapy Providers to Drug and Alcohol Treatment Centers

An illustrative example of the South Carolina program that is in stage 1 of OAN maturity is that of the telehealth-enabled medication-assisted therapy program that connects eligible prescribers to local drug and alcohol treatment centers to treat patients with opioid use disorder. As the need for this clinical service was identified, the key stakeholders met to establish a collaborative forum to discuss the questions listed in Table 2 and apply the OAN standards that had been established by the SCTA technology workgroup. Broadband capability was assessed at each of the drug and alcohol treatment centers that were provided with federally subsidized broadband, when necessary. The program utilizes standards-based video conferencing equipment and software to connect the patient with contracted providers, and technical support is provided by a common SCTA support partner. As the program matures, workgroups are forming to progress toward clinical and administrative consensus.

#### Stage 2: Collaborative Network

Although agreeing upon technical standards is an essential first step, the challenging process of collaboration must be a continuous area of focus to achieve the purposes of the OAN.

##### Ongoing Technical Collaboration

The implementation of a regional telehealth network requires a high degree of collaboration among the participating sites and service providers who may be unaccustomed to external collaborations. The coordination of efforts on a shared vision for a telehealth network is crucial for successful implementation and deployment. Regulatory requirements and threats to IT security create an environment where IT staff are very protective of their organization’s networks. These factors create challenges to establishing trust and working together for the common goal of a statewide health care network. In South Carolina, a three-step process was used to develop and mature statewide IT relationships. In step 1 through the SCTA, we formed an inclusive statewide IT workgroup with representation from members of participating sites. Representation included participation from rural health care providers, community hospitals, regional hospitals, and the state’s largest medical centers. In step 2, the workgroup agreed upon a common vision for the network and developed standards used to implement the network with a focus on protecting existing infrastructure and end point investments. In step 3, key stakeholders worked together to enhance the network by addressing needs for accessing technical support, online directory services for end points, and a shared on-call pool for emergent support needs. Working collaboratively and building trust are the cornerstones for developing and implementing a statewide OAN.

##### Collaborative Design in the Context of Performance and Security

#### Network Security

With federal and state mandates for the protection of health care information, South Carolina designed the OAN to utilize encryption technologies for all video calls. End points across multiple institutions and clinical sites are configured to standards that include required encryption configurations. Laptops utilized in the delivery of telehealth have encryption technologies installed to protect the laptop and its data from unauthorized access. User accounts are needed for access to all PC-based video clients.

#### Network Management

Successful design, implementation, and support of a telehealth network is not complete without a comprehensive network management policy and process. Network management should address procedures for the following:

1. Service level agreements on uptime, trouble resolution, and customer expectations.
2. Asset and inventory management
3. Proactive real-time monitoring of all critical systems
4. Equipment life cycle management
5. Problem escalation procedures
6. Equipment manufacture support agreements
7. Ticketing system for reporting, resolving, and managing trouble calls
8. Knowledgebase for information sharing

##### Clinical Collaboration

Establishing a venue for clinical collaboration is another important, perhaps essential, element of a successful OAN. Even for competing organizations, there is room to collaborate on quality and value alignment care processes. Indeed, the experience in South Carolina has demonstrated that for some services, telehealth can actually help forge partnerships across potentially competing health systems.

This stage was first demonstrated in a partnership between the Medical University of South Carolina (MUSC) and the South Carolina Department of Mental Health. Both entities serve many of the same community hospitals by providing emergency room consultations via telehealth. Although the services and even video end points differed, the two institutions worked together to leverage technology resources and agreed-upon approaches to solving technology problems for the sites they commonly serve. Technical collaboration has matured between the provider groups with common practices both in technology choice and security and network support. Clinical collaboration has been established with one provider group providing care on the inpatient side and another in the emergency department and outpatient setting requiring clinician communication for smooth handoffs.

##### Administrative Collaboration

An important element for the long-term success of a collaborative OAN effort across health systems is leadership buy-in and administrative inclusion. Establishing a mechanism for discussing the administrative elements of contracting, credentialing, scheduling, and business planning while the clinical and technical work is underway can greatly streamline any difficulties faced and lead to synergies in how each participating health system operates in the telehealth space. If possible, having the telehealth network included within the core strategic initiatives and alignment with the health systems mission is ideal for ensuring long-term success of the OAN.

##### Stage 2 Example: Telemental Health Services in South Carolina

Stage 2 of the OAN was first demonstrated in a partnership between the MUSC and the South Carolina Department of Mental Health. Both entities serve many of the same community hospitals by providing emergency room consultations via telehealth. Although the services and even video end points differed, the two institutions worked together to leverage technology resources and agreed-upon approaches to solving technology problems for the sites they commonly serve. Technical collaboration has matured between the provider groups, with common practices both in technology choice and security and network support. Clinical collaboration has been established with one provider group providing care on the inpatient side and another in the emergency department and outpatient setting requiring clinician communication for smooth handoffs. Administrative collaboration has also been ongoing such that leadership finds the relationship so important that this same division of telemental health services that began in community hospitals is now replicated in MUSC’s own children’s hospital as well.

#### Stage 3: Functional Clinical Network

The first two stages of OAN maturity deal with technical and collaborative infrastructure. In stage 3, there is a focus on the demonstration of a functional interoperable network, with different institutions providing services through common telehealth end points at a patient site. To make this transition, there is a focus on distributing the established guidelines to focus on training and smooth *launching* or *go live* of services. Finally, quality metrics are tracked to ensure fidelity and encourage ongoing quality improvement. Attention to this ongoing process acknowledges that the technical, clinical, and administrative and achievements of stages 1 and 2 may not be fully matured and require ongoing attention and modifications. Embracing interoperability will likely lead to scaling of functionality to cost while using common end points. It is in stage 3 where these realities are confronted to move toward stage 4 while not sacrificing clinical integrity.

##### Distribution of Best Practices

For providers at multiple institutions to put the OAN technology to use, there must be a shared understanding of the best practices established by the workgroups during stages 1 and 2. Although all intricacies of the technology choices are not necessarily of utmost importance to this group, a basic understanding of telehealth and how to use and troubleshoot the technology is vital. In addition, when a consensus has been reached to the questions listed in Table 2, a forum for distributing these guidelines to a larger group helps to overcome knowledge barriers. The SCTA has worked through the distribution of instructional information in a variety of ways. Both content advisory and education workgroups exist to help increase general awareness and make more detailed training documents available. These range from a large library of promotional videos to a repertoire of online training modules developed in collaboration with the state’s public television and broadcasting agencies and the South Carolina Area Health Education Consortium. Although these materials are in continued development, their use in conjunction with state and regional group training sets the stage for more in-depth on-site training. These program-specific training for providers and support staff provide employees from multiple institutions with a shared understanding before any go live.

##### Program Go Live

Collaborative use of open access technology by multiple institutions requires substantial coordination and support throughout the process of program development and transition to patient care. This is often best facilitated through dedicated telehealth coordinators or designated contact personnel at each institution. These designees are able to provide a source of continuity between IT, clinical, and administrative teams and host forums for discussion among the institutions. As the training is completed, they are able to host a series of *mock* connections before the rollout of services involving actual patient care and then monitor the first several patient encounters. During this transition period, the coordinators are able to help troubleshoot or find solutions to issues that might otherwise provide a less-than-optimal experience for either the patient or provider.

##### Quality Metric Tracking and Improvement

As in any process improvement effort, establishing metrics of success that can be tracked over time is essential. Ultimately, monitoring successful uses of common end points into the OAN by multiple institutions is a useful metric, but there are likely others needed to obtain this goal. In that stage, the practical realities that inhibit full deployment of the OAN are addressed or at least acknowledged as areas of optimization. These differences in functionality do not exclude the possibility of interoperability, but the clinical and cost needs may understandably be prioritized over interoperability in stage 3. In this stage, these practical realities are acknowledged, and a course is toward interoperability through process improvement and adjustments to technologies.

##### Stage 3 Example: Federally Qualified and Rural Health Centers

As the established infrastructure for providing health care for underserved citizens, many of the federally qualified health centers and rural health centers in South Carolina provide examples of stage 3 OAN maturity. These centers are able to use standards-based equipment to allow for connections to services between their own sites and community-based sites such as schools and drug and alcohol rehab facilities and to receive care from distant sites. They have contributed to several of the workgroups tasked with establishing standards and technology, administrative and clinical infrastructure, and the development of specific clinical programs. Their staff members and providers have taken advantage of common training materials and sessions to learn and help establish best practices. On-site training at these sites and those providing care is facilitated by coordinators, ensuring that all involved are ready for an organized go live. Patient care at these sites may utilize different workflows; however, the use of standards-based equipment and common practices, enable efficient use of technology. Finally, quality metrics are beginning to be established at these clinics to track and facilitate the continuous improvement of patient care.

#### Stage 4: Standard Process Network

Once a use case of two institutions using a common end point has been demonstrated, the transition to stage 4 maturity is underway. In this stage, optimization of the network focuses on the elements that will truly make the OAN add value to the regional care system. In this stage, clinical workflows can be standardized across institutions, and economies of scale can be achieved through administrative and technical innovations.

##### Establishment of Common Workflows

With the standardization of clinical workflows, there is a common experience of the clinical providers and staff, allowing for an elevation of skill level and generalization of knowledge and competencies. Administrative innovations may include common scheduling portals, standardization of contract language, and streamlining of credentialing needs. From a technical perspective, optimization may include the use of software-assisted functionality that complies with interoperability standards. Technical response teams are now highly coordinated with open lines of communication.

##### Shared Administrative and Clinical Resources

A common Web portal for the OAN could be instrumental in bringing many of the aforementioned elements together. However, to remain open access, the costs of the network should remain reasonable for broad participation and not require the use of scaled functionality for all services. Willing partners should not be excluded from the network within the bounds of the agreed-upon standards and compliance with established workflows and procedures. Uniquely, at this level of collaboration, partnerships on service are enabled, in which otherwise competing institutions may find themselves collaborating with shared resources, such as common physician call pools when providers are in short supply.

##### Payer Advocacy

At the most mature stage of maturity, programs are able to reach a scale in which utilization and quality metrics are well established. After some time, at-scale health outcomes and cost efficiencies are also able to be measured. Multiple provider groups are then able to leverage these data to advocate for improved reimbursement either through legislation or direct negotiation with individual payers. Although some programs may ultimately rely on multiple funding mechanisms to ensure sustainability, a collaborative approach to demonstrating the value of telehealth programs and ultimately improving the reimbursement landscape is beneficial to all involved.

##### Stage 4 Example: School-Based Telehealth

The South Carolina school-based telehealth program is an illustrative example of a statewide telehealth program that has moved through each OAN stage of maturity. Early in this program’s development, a commitment was made to adhere to open access standards when selecting technology, and broadband capability in rural schools was established with the help of the FCC-funded PSPN. As multiple health systems and provider groups became involved, the school-based telehealth workgroup within the SCTA was established to provide a framework for team building and sharing of ideas related to both clinical and technological standards. A collaborative IT support network was essential to the growth of this program as relationship building between school and provider IT teams was fundamental to both establishing and maintaining connectivity. Eventually, it became evident that a tiered call pool approach in which a school nurse’s request for a visit could be sent first to a local provider, then to a provider group at the most local regional health care system, and then to a group at the largest state medical system was the best strategy to keep care local while still providing a quick response. For this to become a reality, a common clinical and administrative support network was established. This model has most recently been implemented using a common software platform that can be accessed by multiple end points that meet the open access standards. Although this platform has not become a fully statewide portal, its use has encouraged common workflows and processes, and with increased collaboration, the program has increased efficiency and utilization. Most recently, data have shown that the program may be associated with more cost-effective health care utilization patterns, and the provider groups are working together to advocate for improved reimbursement policies from a variety of payers.

#### Exception to the Rule: Intentional Fragmentation of an Open Access Network

Interoperability in telehealth solutions is often a goal rather than a full reality for most health systems. Although this is true for many health care IT solutions, it is compounded with telehealth because of the realities of different functionalities needed for different clinical situations. Health systems and telehealth vendors are often in diligent pursuit of an *enterprise solution*, though modifications and exceptions to the standardizations in place remain common for certain clinical services. Embracing the idea that alternative technologies may be needed to achieve clinical goals can be helpful if it allows for a focus on prioritizing what exceptions and options are tolerable, and to work on the integrations needed. This prioritization can be seen as being intentional with your telehealth systems’ fragmentation. Areas of prioritization to consider when faced with conflicting telehealth technology needs include the following:

1. Clinical functionality
2. Delays in service delivery pending prolonged integrations
3. Functionalities that add efficiencies not present in a health system’s legacy workflows
4. Cost
5. End user experience, including the patient

Once a set of priorities are established, and some fragmentation of the telehealth technology ecosystem is allowed, it should also be the goal of eventual full standardization. Working toward a system with smooth user experience, maximized functionality, and optimal cost may require long-term goal setting and shared decision making across varying levels of health systems governance.

#### Examples of Intentional Fragmentation From the South Carolina Open Access Network

##### Example 1 of Deviation From the Open Access Network: Telestroke

Telestroke, which is a highly successful use of telehealth across the country, is an example of successful clinical collaboration on common technology that is not tied to the South Carolina OAN. The operational needs of this type of program include on-demand call access to providers and rapid integration of radiological imaging, which often leads to the use of niche, proprietary software apps, as it did here in South Carolina. This telestroke network has led the way in terms of clinical collaboration, with multiple health systems contributing to a common pool of providers for the good of the region and sustainability of the service. However, integrating the OAN with this high-volume, high-stakes service remains a challenge, and thus intentional fragmentation of the OAN permitted.

##### Example 2 of Deviation From the Open Access Network: Tele-Intensive Care Unit

One example of a telehealth program that is unlikely to meet even the most basic OAN standards are the most widely used technologies for tele-intensive care units (tele-ICUs). Tele-ICU, as conventionally defined, offers continuous remote patient monitoring from a multidisciplinary clinical ICU team led by an intensivist in a fixed space operations center. Each ICU patient room being monitored is wired with two-way audiovisual communication technology, and an emergency alert button is installed that can be activated during patient crisis. The most common technology for this clinical application includes proprietary software that enables a centralized patient census along with easy access to the audiovisual communication system. The two-way communication system is controlled by the remote clinicians and is supported by a separate vendor from the tele-ICU system. Some clinical information derived from the EHR is included in the centralized system, although this varies across programs and is often limited to basic electronic data provided from discreet data fields in the EHR (eg, patient vital signs and laboratory testing). A tele-ICU clinician workstation in the operations center includes 8 to 12 separate monitors and has numerous technologic interfaces established between the operations center and the remote ICU, including EHR interfaces, physician order entry interfaces, bedside monitor waveform interfaces, radiology viewing interfaces, and internal communication systems for operations center personnel.

## Discussion

Telehealth is a rapidly growing component of the US health care system. Yet, to achieve large-scale adoption, policy makers and health care stakeholders should consider options that cross the traditional boundaries of proprietary health care marketplaces. The OAN is one approach that facilitated the statewide telehealth network in South Carolina.

The approach to OAN development described here provides a roadmap for achieving a functional telehealth network across independent health systems. The South Carolina experience reveals both successes and challenges in achieving this goal. The next steps toward the development of OANs would include advocacy and ongoing engagement with the developers of telehealth technologies regarding their commitment to interoperability. In addition, focus on health system partnerships, and collaborative processes round out the essential elements of the OAN.

## Abbreviations

EHR: electronic health record
FCC: Federal Communications Commission
HHS: Health and Human Services
HRSA: Health Resources and Services Administration
ICU: intensive care unit
IT: information technology
ITU: International Telecommunications Union
MUSC: Medical University of South Carolina
OAN: open access network
PSPN: Palmetto State Providers Network
SCTA: South Carolina Telehealth Alliance
Tele-ICU: tele-intensive care unit
